# Mapping Knowledge Landscapes and Emerging Trends of the Links Between Bone Metabolism and Diabetes Mellitus: A Bibliometric Analysis From 2000 to 2021

**DOI:** 10.3389/fpubh.2022.918483

**Published:** 2022-06-03

**Authors:** Kunming Cheng, Qiang Guo, Weiguang Yang, Yulin Wang, Zaijie Sun, Haiyang Wu

**Affiliations:** ^1^Department of Intensive Care Unit, The Second Affiliated Hospital of Zhengzhou University, Zhengzhou, China; ^2^Department of Orthopaedic Surgery, Baodi Clinical College of Tianjin Medical University, Tianjin, China; ^3^Graduate School of Tianjin Medical University, Tianjin, China; ^4^Department of Orthopaedic Surgery, Clinical College of Neurology, Neurosurgery and Neurorehabilitation, Tianjin Medical University, Tianjin, China; ^5^Department of Orthopaedic Surgery, Xiangyang Central Hospital, Affiliated Hospital of Hubei University of Arts and Science, Xiangyang, China

**Keywords:** diabetes mellitus, bone metabolism, bibliometrics, CiteSpace, VOSviewer, Bibliometrix

## Abstract

**Background:**

Diabetes mellitus (DM) have become seriously threatens to human health and life quality worldwide. As a systemic metabolic disease, multiple studies have revealed that DM is related to metabolic bone diseases and always induces higher risk of fracture. In view of this, the links between bone metabolism (BM) and DM (BMDM) have gained much attention and numerous related papers have been published. Nevertheless, no prior studies have yet been performed to analyze the field of BMDM research through bibliometric approach. To fill this knowledge gap, we performed a comprehensive bibliometric analysis of the global scientific publications in this field.

**Methods:**

Articles and reviews regarding BMDM published between 2000 and 2021 were obtained from the Web of Science after manually screening. VOSviewer 1.6.16, CiteSpace V 5.8.R3, Bibliometrix, and two online analysis platforms were used to conduct the bibliometric and visualization analyses.

**Results:**

A total of 2,525 documents including 2,255 articles and 270 reviews were retrieved. Our analysis demonstrated a steady increasing trend in the number of publications over the past 22 years (*R*^2^ = 0.989). The United States has occupied the leading position with the largest outputs and highest H-index. University of California San Francisco contributed the most publications, and Schwartz AV was the most influential author. Collaboration among institutions from different countries was relatively few. The journals that published the most BMDM-related papers were *Bone* and *Osteoporosis International*. Osteoporosis and related fractures are the main bone metabolic diseases of greatest concern in this field. According to co-cited references result, “high glucose environment,” “glycation end-product” and “sodium-glucose co-transporter” have been recognized as the current research focus in this domain. The keywords co-occurrence analysis indicated that “diabetic osteoporosis,” “osteoarthritis,” “fracture risk,” “meta-analysis,” “osteogenic differentiation,” “bone regeneration,” “osteogenesis,” and “trabecular bone score” might remain the research hotspots and frontiers in the near future.

**Conclusion:**

As a cross-discipline research field, the links between bone metabolism and diabetes mellitus are attracting increased attention. Osteoporosis and related fractures are the main bone metabolic diseases of greatest concern in this field. These insights may be helpful for clinicians to recognize diabetic osteopenia and provide more attention and support to such patients.

## Introduction

Diabetes mellitus (DM) is a chronic metabolic disease, characterized by disturbances in insulin and glucose metabolism. According to the estimates from World Health Organization (WHO), DM, especially type 2 DM (T2DM), affects 8.5% of world's total population aged 18 years or older, and it will become the 7th leading cause of death worldwide by 2030 (1). DM is able to cause damages to a variety of organs, in particular, nervous system, kidneys, and blood vessels. Substantial evidence suggests that a longer exposure to hyperglycemia also make an impact on bone metabolism (BM) (2, 3). Initially, many clinicians observed that diabetic patients had a higher risk of fractures as well as impaired fracture healing. Until 2007, a meta-analysis conducted by Vestergaard (4) reported for the first time that hip fracture risk was significantly increased in both type 1 DM (T1DM) and T2DM. And compared to subjects without DM, the risk of hip fracture was 6.94 and 1.38 times higher in T1DM and T2DM individuals, respectively. Apart from hip fracture, similar conclusions were reached from several other large sample meta-analyses on the association between DM and fracture risk at other sites including vertebra, upper arm, distal forearm, and ankle (5–7). Since then, osteoporosis and related fractures are the main bone metabolic diseases of greatest concern in this field (8, 9). Many experts have been calling attention to the osteoporosis/bone loss management and fracture risk assessment for DM patients. In 2019, several scholars from the Chinese Medical Association (CMA) even developed a joint expert consensus for the fractures risk management in DM patients (10).

In the light of this, the links between BM and DM (BMDM) have gained widespread attention from researchers recently. A large aggregation of studies devoted to deciphering the complex mechanisms about how diabetes works on bone remodeling (2). To date, despite more than 20 years of intensive research and several hypotheses, such as oxidant injury, accumulation of advanced glycation end products, increased collagen glycation, bone-fat axis hypothesis, and some other intermediate mediators including hormones, cytokines and nutrients, have been proposed, the specific mechanism of diabetes-related reduction of bone mass remains unclear (11–13). In addition, with the development of novel antidiabetic agents, it has been reported that various glucose-lowering medications including thiazolidinedione, insulin, metformin, sodium glucose co-transporter 2 inhibitors, dipeptidyl peptidase-4 (DPP-4) inhibitors have different influences on bone formation and resorption (14–18).

Motivated by the above concerns, numerous papers related to BMDM have been published. However, in the face of massive information in this topic, researchers need to dedicate a great deal of time to reading and understanding appropriate work in interrelated disciplines. Hence, it is imperative to classify substantial, classical, and evocative evidence to assist the scientific investigation (19). Although several literature reviews and meta-analyses are able to provide useful information and reliable evidence-based medical findings, these methods are often unable to make a summary from a holistic and integrated perspective for a specific field of research (20, 21).

Bibliometric analysis applies statistical and mathematical approaches to quantitatively and qualitatively analyze all carriers of knowledge such as journal articles. It is an important and well-established method to spot active researchers and potential collaborators, sort out hot topics, describe the dynamic trends, and identify future research frontiers (22, 23). Recently, bibliometric analysis and data visualization have received a considerable amount of attention in biomedical fields owing to the explosion in basic and clinical data, and the growing number of freely available bibliometric tools over the past few years (24, 25). Taking DM and metabolic bone disorders as examples, multiple investigators have studied the scientific outputs and publication trends of diabetes-related research across different countries via bibliometric approaches (26–28). DM-associated complications such as diabetic foot ulcers (29), diabetic nephropathy (19, 30), and diabetic ophthalmopathy (31), etc. have also been studied with this approach. Our and other research teams have also investigated the publication status and research hotspots in the field of metabolic bone disorders including osteoporosis (32), hip fracture (33), osteonecrosis (34), and osteoarthritis (35) by using bibliometric methods, and mapped the overall knowledge structures and co-citation networks in these areas. Nevertheless, to the best of our knowledge, there are still no previous bibliometric studies reporting the links between BM and DM.

As such, we sought to fill this knowledge gap. In this study, multiple software programs and online platforms were used to analyze BMDM-related literature, as well as draw the scientific knowledge maps. The primary aims of the study were to (i) identify the main contributors including authors, institutions, and countries in the BMDM field from 2000 to 2021; (ii) explore the development and evolution trend of research focus; (iii) predict future research frontiers of this area; (iv) offer some new perspective and ideas for the subsequent studies between diabetes and bone metabolism; (v) call for more attention, especially clinician and researcher on this subject.

## Materials and Methods

### Data Source

The Web of Science Core Collection (WoSCC, Clarivate Analytics, Philadelphia, PA, USA) is one of the most professional and authoritative citation-based databases with powerful indexing functions, which not only contains the basic information including titles, authors, institutions, countries/regions, and author keywords, but particularly includes the references information (22, 23). Therefore, it is considered as the optimal database and widely used in previous bibliometric studies (36). In this study, we selected to retrieve publications related to BMDM in the WoSCC of Science Citation Index Expanded (SCIE).

### Data Search Strategy

**Figure 1:**
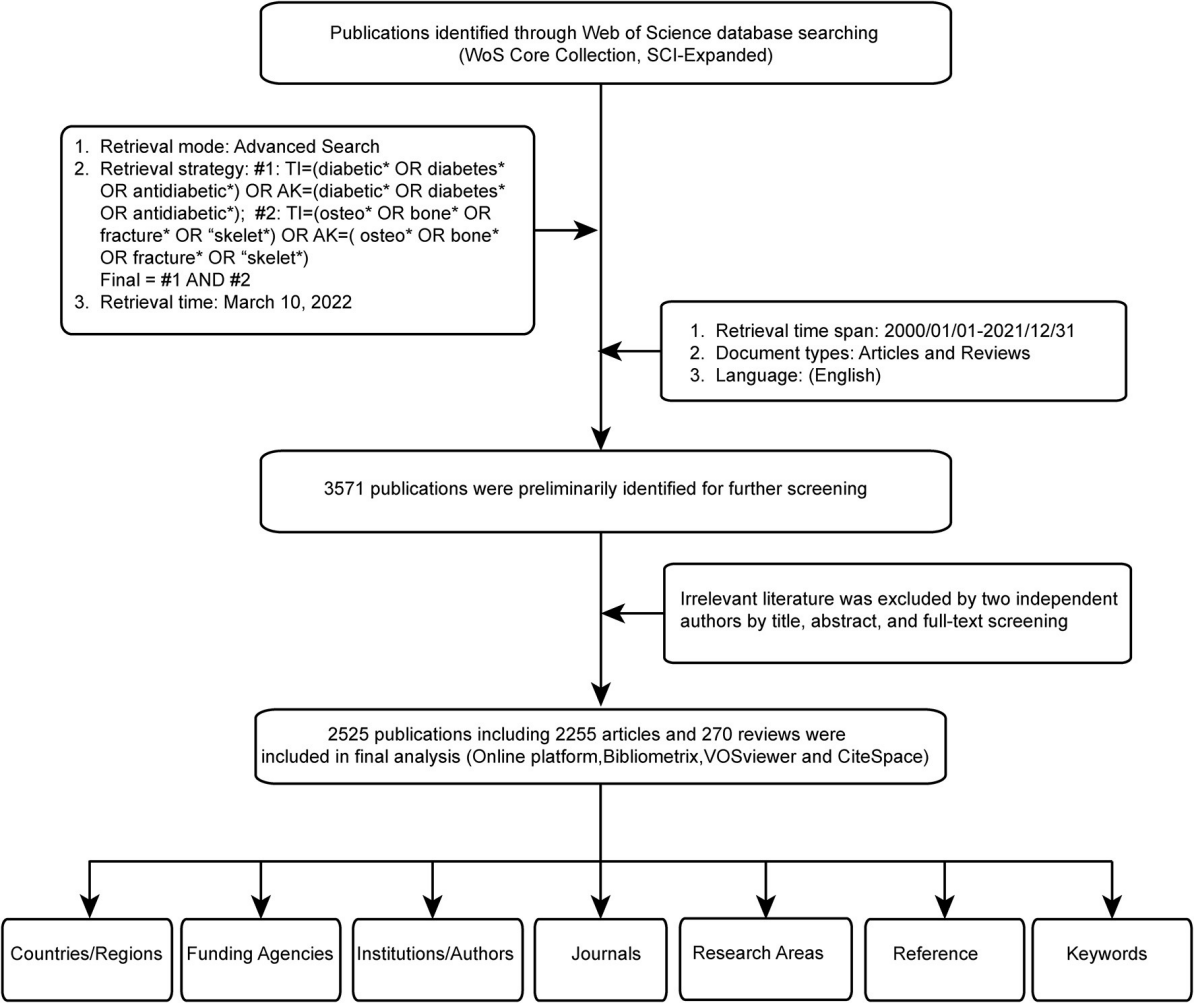
Flowchart of literature search and selection process.

Considering that the database is still functioning and may update daily, a comprehensive online search was performed on a single day by two authors (CK and WH), to avoid the bias. All potentially relevant publications were collected based on the titles (TI) and author keywords (AK) with the following search formula: #1: [TI = (diabetic^*^ OR diabetes^*^ OR antidiabetic^*^) OR AK = (diabetic^*^ OR diabetes^*^ OR antidiabetic^*^); #2: TI = (osteo^*^ OR bone^*^ OR fracture^*^ OR “skelet^*^) OR AK = (osteo^*^ OR bone^*^ OR fracture^*^ OR “skelet^*^); Final dataset: #1 AND #2]. In order to achieve as many relevant sources as possible, a wildcard character (^*^), representing one or more other characters and allowing variable endings of keywords was used. For example, osteo^*^ would also return the terms of osteoporosis, osteopenia, osteoporotic, osteoarthritis, and so on. Then, a timespan of 22 years was set and only studies published from 2000 to 2021 were included. The literature language was restricted to English. The literature types were limited to articles and reviews, with the specific exclusion criteria shown in Figure 1. A total of 3,571 documents were retrieved as potential candidates for inclusion from the initial literature search. Then the literature titles, abstracts, and the full text were manually examined by two investigators (CK and GQ) to exclude literature that was irrelevant to study topic (including disease type, research purposes, interventions, outcome indexes, etc.). A total of 2,525 articles and reviews were included for data analysis after the final selection.

### Data Extraction and Collection

All the 2,525 retrieved literature was downloaded with “full record and cited references” and exported in plain text or tab-delimited (win, UTF-8) format for the analysis of bibliometric tools. Subsequently, Microsoft Excel 2019 was used for statistical analysis of the bibliometric indicators including annual number of publications and citations, countries/regions, institutions, authors, funding agencies, journals, keywords, as well as research areas. Some inherent deficiencies from the WOS database were checked and merged, and information from various regions was incorporated into their affiliated countries. For example, publications from England, Northern Ireland, Scotland, and Wales were assigned to the UK. The journal impact factor (JIF) and subject category quartile ranks were obtained from the 2020 Journal Citation Report (JCR, http://clarivate.com/products/web-of-science). Based on the value of JIF, JCR splits all the journals within the same discipline into four categories, of the top 25% belonging to Q1 and the top 25–50% being Q2, and so on. Some other bibliometric information including sum of time cited, average number of citations, and H-index was acquired from the “citation report” function of WoSCC.

### Bibliometric Analysis

In order to obtain more comprehensive data analysis, three bibliometric software including VOSviewer 1.6.16 (Leiden University, the Netherlands), CiteSpace V 5.8.R3 (Drexel University, the USA), Bibliometrix (University of Naples Federico II, Italy), and two online analysis platforms were used for bibliometric and visualization analyses.

VOSviewer, a free java-based software for bibliometric mapping and clustering analysis, was developed by van Eck and Waltman (24). In this study, country/institution/author co-authorship analysis, author/journal co-citation analysis and keyword co-occurrence analysis were conducted in this study. Generally speaking, in these visual maps, different nodes indicated different items such as authors, countries, institutions, journals and keywords, with the nodes size reflecting the corresponding number of publications citations or occurrences. The links between nodes represented the co-authorship, co-citation or co-occurrence associations between nodes. The color of the nodes and lines indicated different clusters or corresponding average appearing year (AAY) (23, 37).

CiteSpace, developed by Professor Chen Chaomei, is another software tool for visualizing and constructing bibliometric networks (25). In this study, CiteSpace was used to visualize international collaboration among institutions, the co-citation of references and its cluster analysis. We also identified several references that experienced the greatest increase in the citation frequencies over a certain period, which was considered a period of popularity for the study. The parameters of CiteSpace were set as follows: time span (2000–2021), years per slice (1), selection criteria (Top 30), and pruning (minimum spanning tree, pruning sliced networks).

In addition, the online bibliometric analysis platform, available at: https://bibliometric.com/, was used to conduct the collaboration networks between countries, and annual publication trend analysis of the top three most productive countries (38). We also used Bibliometrix to perform descriptive analysis of the authors' production over time and thematic evolution of keywords. Furthermore, gene-based analysis of osteoporosis or osteoarthritis in diabetes mellitus was analyzed by the online website (https://www.citexs.com/Summary). This website allowed us to summarize the gene data of all the studies in a certain field, and the publications were acquired based on Pubmed database.

### Statistical Analysis

Descriptive data analysis, graph plotting and curve fitting were performed with Microsoft Excel 2019, R software (v 4.1.0) and GraphPad Prism software (v 8.0). The annual number of publications and citations were calculated with Microsoft Excel, and the following types of functions including exponential, linear, logarithmic, and polynomial were used to fit curves. The best-fit model was selected according to the magnitude of the correlation coefficient (*R*^2^). The growth rate of publications over time was calculated based on the specific calculation formula as follows (23): Growth rate = [(number of publications in the last year ÷ number of publications in the first year)^1/(lastyear−firstyear)^−1] × 100. Pearson's correlation coefficient test was used to evaluate the correlation between publications and citations, and Pearson correlations corresponding to a *p*-value < 0.05 were considered significant.

## Results

### Publication Outputs and Trends

**Figure 2:**
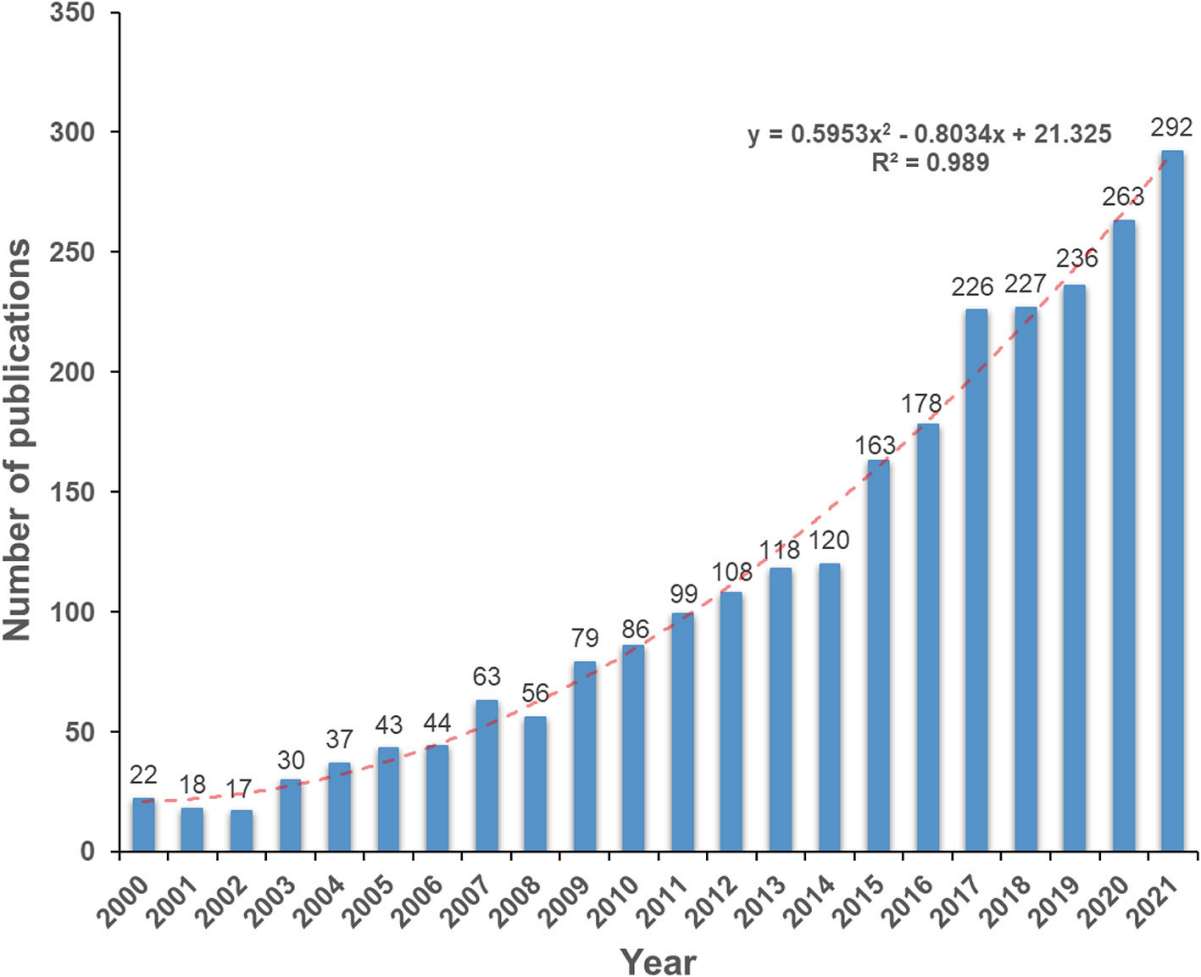
The specific amount of annual publications regarding BMDM from 2000 to 2021.

Based on the literature search and screening strategy in Figure 1, a total of 2,525 literature studies including 2,255 (89.3%) articles and 270 (10.7%) reviews were finally identified. Figure 2 depicts the specific amount of annual publications regarding BMDM. From 2000 to 2021, the average growth rate of publications was 13.1%. Moreover, in order to further evaluate the change trend of BMDM studies, the index function Y = 0.5953X^2^-0.8034X + 21.325 (*R*^2^ = 0.989, X is the year, Y is the annual publications) of the annual publication trend was created. When it comes to the annual number of citations, it also showed a similar increasing trend (*R*^2^ = 0.9974) as the annual publication number (Supplementary Figure 1). Upon plotting the correlation between publications and citations, a statistically significant linear correlation was observed with a satisfactory Pearson's correlation coefficient (*r* = 0.996) and explicability (*R*^2^ = 0.992). Thus, the variations in the citation rate can mostly be explained by the publication rate.

### Most Productive Countries/Regions and Funding Agencies

**Figure 3:**
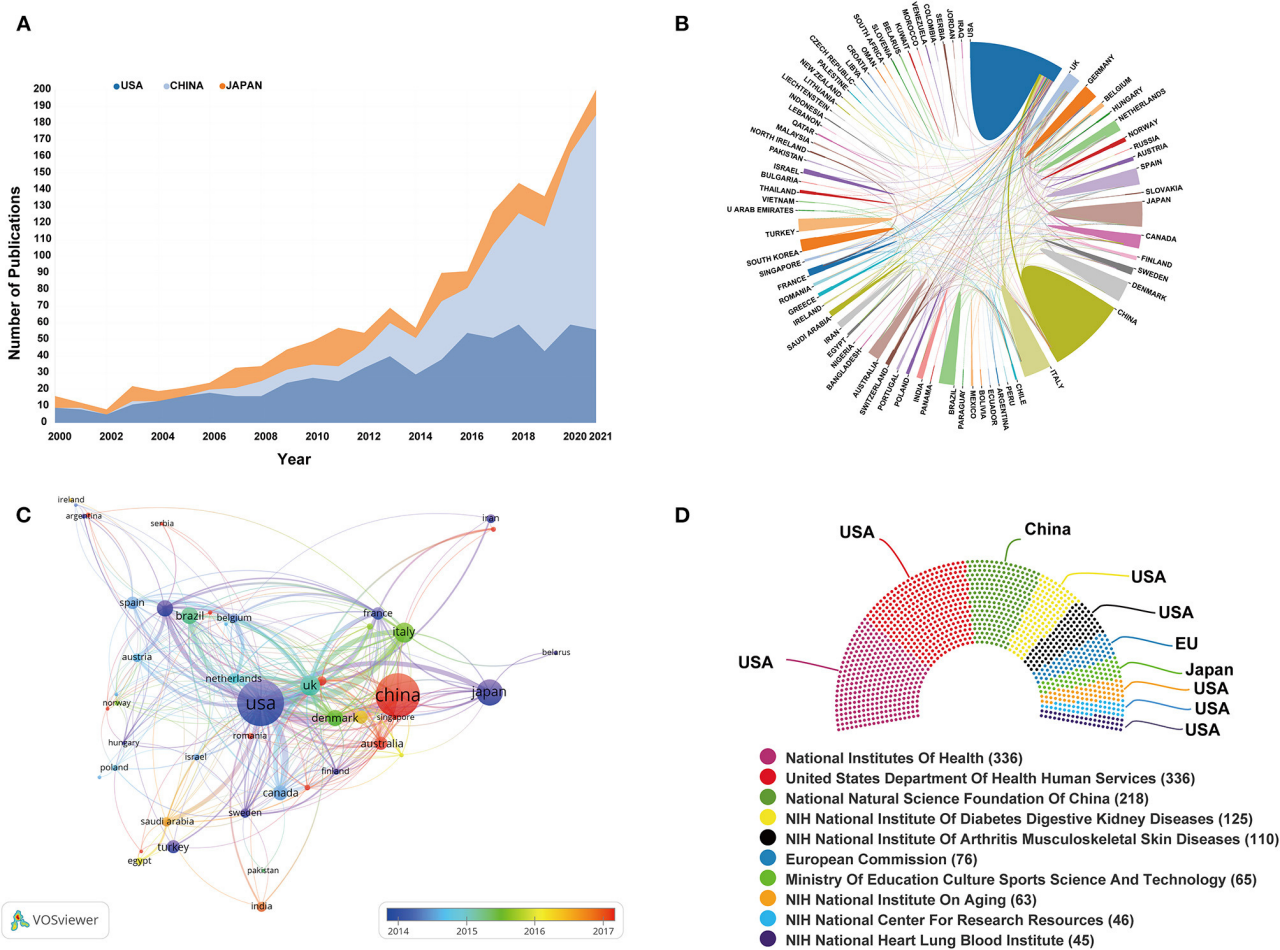
**(A)** The annual publications of the top 3 countries from 2000 to 2021. **(B)** The international cooperation analysis among different countries. Line thickness between countries reflects the intensity of the closeness. **(C)** The overlay visualization map of country co-authorship analysis conducted by VOSviewer. Each country is represented as a node, and the node size is proportional to the sum of publications. A link between two nodes indicates a co-authorship relationship. Different nodes were given different colors (based on AAY) according to the color gradient in the lower right corner. **(D)** The top 10 most frequent funding sources in this field.

A total of 78 countries/regions contributed all publications on BMDM research. As can be seen from Table 1, the most prolific country is the United States (*n* = 656, accounting for 25.98% of the total), followed by China and Japan. Figure 3A summaries the annual publications of these top 3 countries from 2000 to 2021. Among the top 20 most productive countries, the United States has the highest H-index of 97, and far higher than other countries/regions. Nevertheless, based on the average citations per document, the ranking of countries is: Netherlands (60.2), the United States (50.2), and Canada (49.37). Figure 3B displays the international cooperation analysis among different countries. As shown in Figure 3C, the overlay visualization map of country co-authorship analysis was conducted by VOSviewer. Literature originating from 47 countries/regions was selected, with the minimum number of 5 documents for each country. The United States was situated in a central position of this network map. Figure 3D summarizes the data of the top 10 most frequent funding sources in this field, with seven funding agencies based in the United States.

**Table 1:** Top 20 most productive countries in the research field of BMDM.

**Ranking**	**Countries**	**Publications, n**	**% of 2,525**	**H-index**	**Average citations per document**
1	USA	656	25.98	97	50.2
2	China	596	23.60	39	12.6
3	Japan	240	9.50	48	30.9
4	UK	151	5.98	45	39.13
5	Italy	146	5.78	38	33.58
6	Brazil	107	4.24	26	18.24
7	Germany	103	4.08	35	39.31
8	Denmark	99	3.92	30	46.79
9	Canada	86	3.41	30	49.37
10	South Korea	71	2.81	17	20.27
11	Australia	69	2.73	20	20.25
12	Spain	67	2.65	22	21.97
13	Turkey	67	2.65	22	17.97
14	France	66	2.61	28	38.8
15	Netherlands	49	1.94	24	60.2
16	India	43	1.70	14	14.93
17	Sweden	41	1.62	17	38.12
18	Saudi Arabia	39	1.54	14	17.21
19	Belgium	38	1.50	19	23.97
20	Switzerland	37	1.47	21	45.89

*Ranking: based on the number of total publications*.

### Analysis of Institutional Output

**Figure 4:**
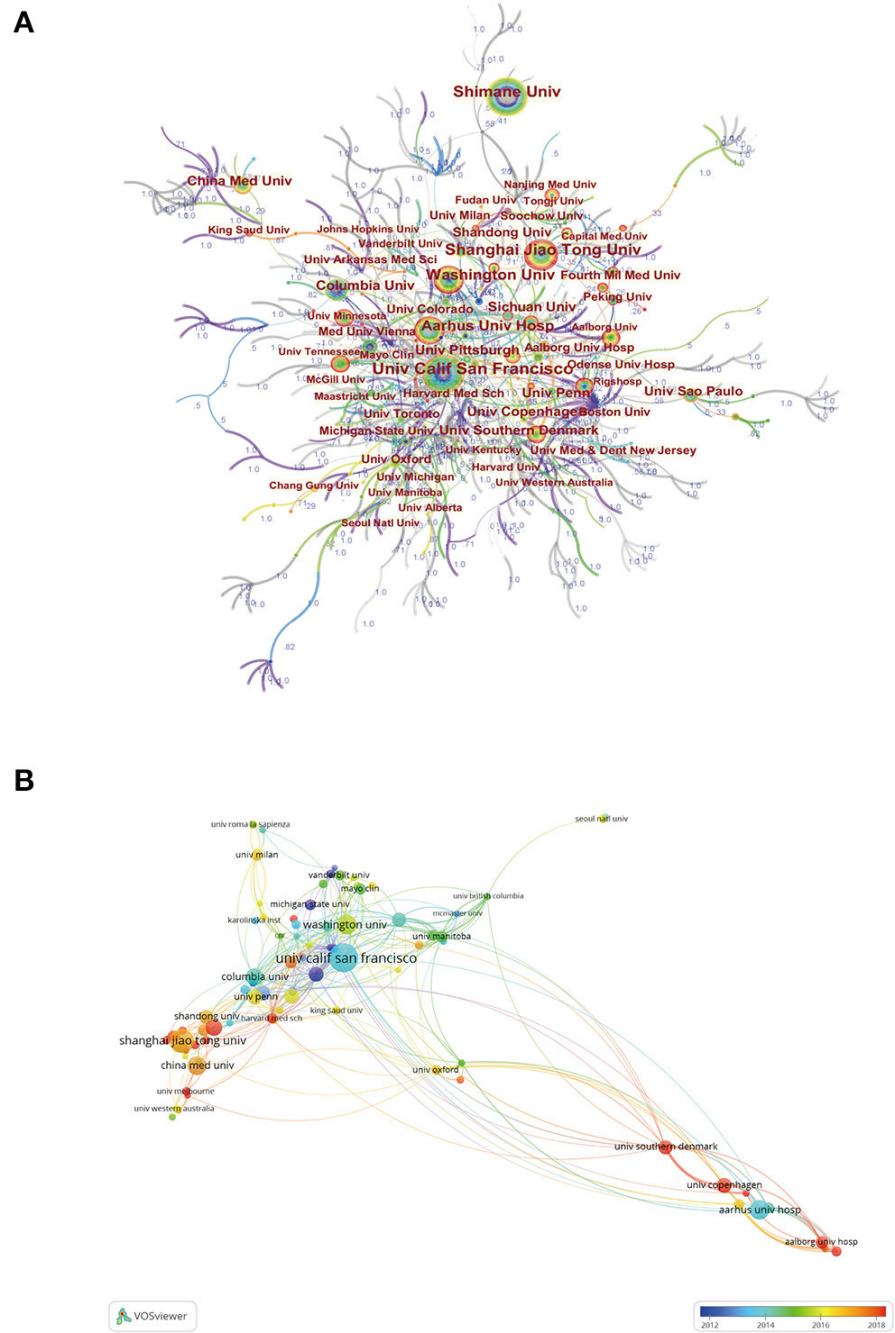
**(A)** Network visualization map of institutional collaborations generated by CiteSpace. In this map, a node represents an institution, and the size of each node represents its relative quantity of research output. Each line represents the strength of the cooperation relationship between two institutions, and strength value is displayed between lines. **(B)** The overlay visualization map of Institution co-authorship analysis conducted by VOSviewer.

A network visualization map of institutional collaboration was generated by CiteSpace and presented in Figure 4A. The top 5 institutions addressed in the largest number of publications were University of California San Francisco, Shanghai Jiao Tong University, Shimane University, Washington University, and Aarhus University Hospital. However, there were relatively few collaborations among institutions from different countries. And only three institutions including University of California San Francisco, Columbia University, and University of Toronto had a betweenness centrality (BC) value more than 0.1. Additionally, institution co-authorship analysis was conducted by VOSviewer (Figure 4B). According to the color gradient in the lower right corner, these institutions such as Aarhus University Hospital, Sichuan University, University of Southern Denmark, University of Copenhagen, etc. were given a red color with the larger AAY values. And corresponding to this, University of Pittsburgh, Michigan State University, University of Minnesota were given a blue color with the smaller AAY values.

### Analysis of Influential Authors

**Figure 5:**
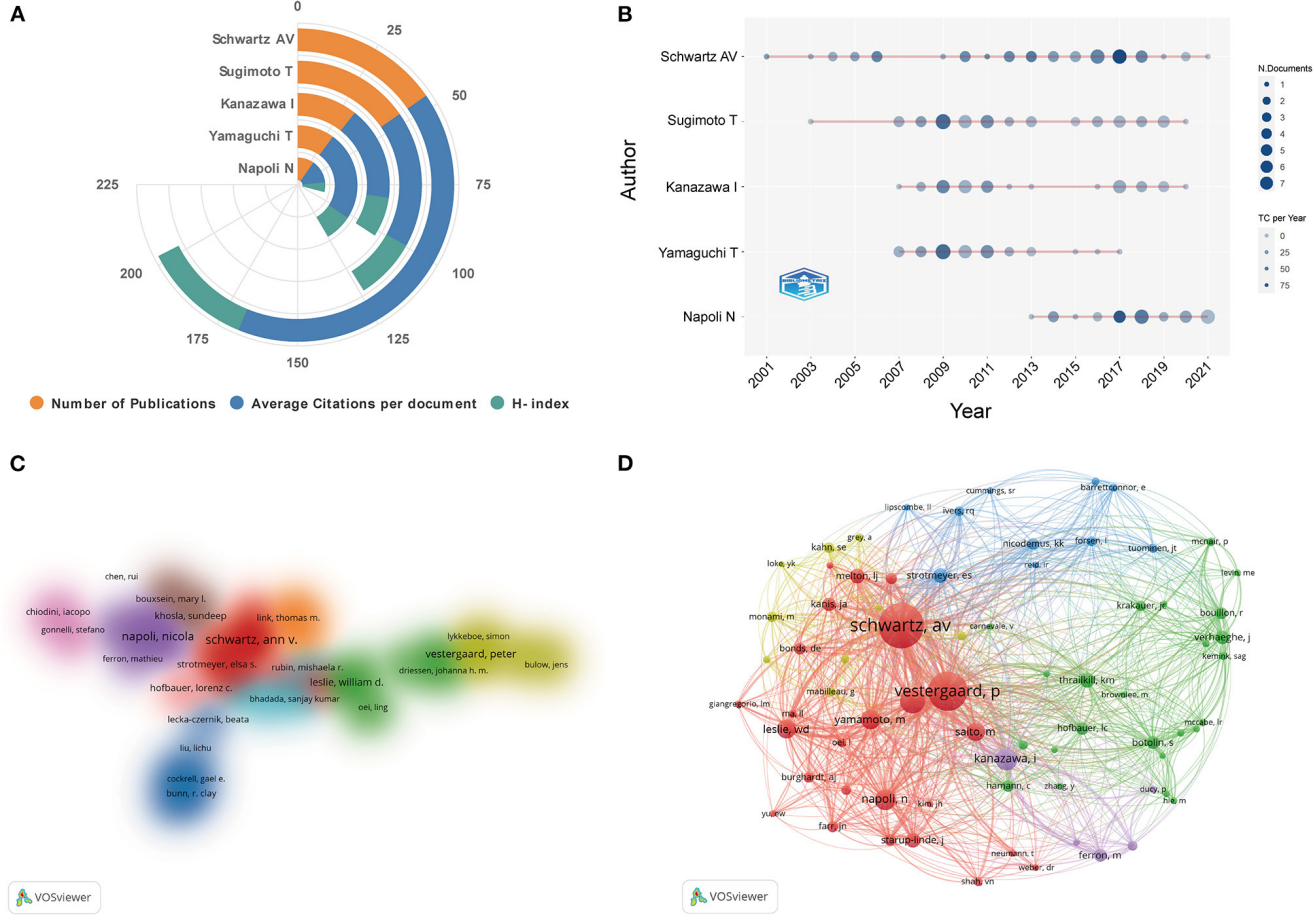
**(A)** Total number of publications, average citations per document, and H-index of top 5 authors in this area. **(B)** Top 5 authors' production over time. The circle size represents the number of documents (N. Documents), and the shade of the color signifies the total number of citations (TC). **(C)** The cluster density map of author co-authorship analysis generated by VOSviewer. Authors with close cooperation relationship are assigned to one cluster with the same color. **(D)** The network visualization map of author co-citation analysis generated by VOSviewer. Each author is represented as a node, and the node size is proportional to the sum of citations. A link between two nodes indicates a co-citation relationship. The distance between nodes indicates the relatedness, and a smaller distance implies a higher relatedness and will be assigned to one cluster with the same colors.

In terms of author analysis, the top 5 most prolific authors were laid out in Figure 5A. Schwartz AV contributed the highest number of publications, followed by Sugimoto T and Kanazawa I. Schwartz AV was also the author with the highest H-index and average citations per document. Figure 5B depicts the annual outputs and citations of the top 5 authors between 2000 and 2021. A cluster density map of author co-authorship analysis is shown in Figure 5C. Only authors with more than 5 documents were included. Of these, a total of 11 author clusters were formed. As for author co-citation analysis, 73 authors with at least 100 citations were included. As displayed in Figure 5D, the top 3 authors with the greatest total link strength (TLS) were Schwartz AV, Vestergaard P, and Janghorbani M.

### Most Active Journals and Research Areas

**Figure 6:**
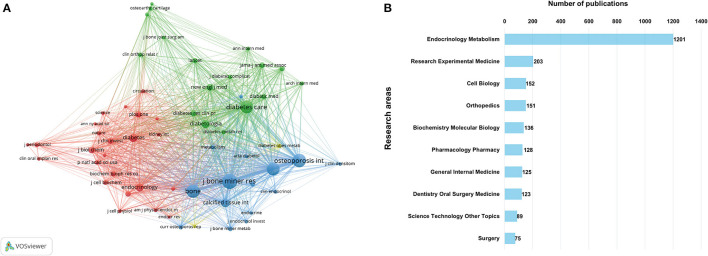
**(A)** Network visualization map of journal co-citation analysis generated by VOSviewer. **(B)** Top 10 research areas ranked by publication counts.

Table 2 shows the basic information of the top 20 most productive journals in BMDM field. Among them, *Bone* has published the greatest number of 122 papers, followed by *Osteoporosis International, Journal of Clinical Endocrinology Metabolism, Calcified Tissue International*, and *Journal of Bone and Mineral Research*. More than four fifths of the top 20 journals were categorized in Q1 or Q2 JCR region. As indicated in Figure 6A, the network visualization map of journal co-citation analysis was further performed by VOSviewer. Only journals with a minimum of 300 citations were visualized. Of the 58 journals satisfying the criteria, the top 5 co-cited journals were *Journal of Bone and Mineral Research, Osteoporosis International, Bone, Journal of Clinical Endocrinology Metabolism, Diabetes Care*. Additionally, according to WoS subject categories, all these literatures are assigned to different research areas. Top 10 research areas ranked by publication counts are exhibited in Figure 6B.

**Table 2:** Top 20 most productive journals in BMDM field.

**Ranking**	**Sources title**	**Output**	**% of 2,525**	**JIF 2020**	**JCR quartile 2020**
1	Bone	122	4.83	4.398	Q2
2	Osteoporosis International	102	4.04	4.507	Q2
3	Journal of Clinical Endocrinology Metabolism	73	2.89	5.958	Q1
4	Calcified Tissue International	56	2.22	4.333	Q2
5	Journal of Bone and Mineral Research	55	2.18	6.741	Q1
6	Diabetes Care	53	2.10	19.112	Q1
7	PLoS One	43	1.70	3.24	Q2
8	Diabetes Research and Clinical Practice	41	1.62	5.602	Q1
9	Journal of Bone and Mineral Metabolism	33	1.31	2.626	Q3
10	Frontiers in Endocrinology	28	1.11	5.555	Q1
11	Journal of Diabetes and Its Complications	28	1.11	2.852	Q3
12	Current Osteoporosis Reports	26	1.03	5.096	Q2
13	Diabetic Medicine	26	1.03	4.359	Q2
14	Scientific Reports	26	1.03	4.38	Q1
15	Acta Diabetologica	23	0.91	4.28	Q2
16	Diabetes Metabolism Research and Reviews	21	0.83	4.876	Q2
17	Endocrine	21	0.83	3.633	Q3
18	Journal of Periodontology	21	0.83	6.993	Q1
19	Diabetologia	20	0.79	10.122	Q1
20	European Journal of Endocrinology	19	0.75	6.664	Q1

*Ranking: based on the number of total publications*.

### Co-cited References and Reference Burst

**Figure 7:**
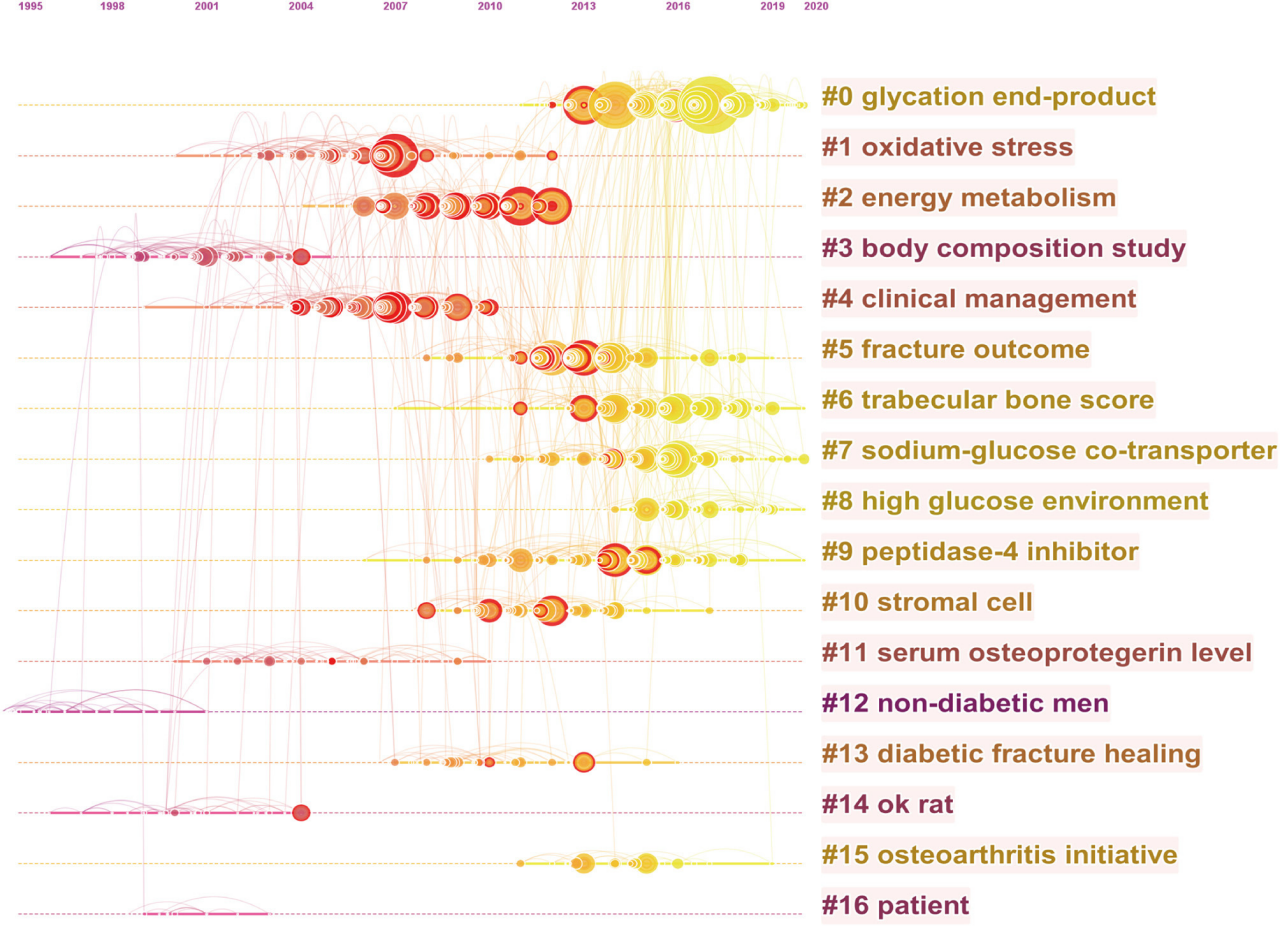
The timeline view map of reference co-citation analysis generated by CiteSpace. Years from 2000 to 2021 are arranged horizontally at the top, and this view map clearly implies the differences in the appearance time point of 17 clusters. The bigger the circle size, the more research on the topic. The label of each cluster (LLR algorithm) is shown at the end of the timeline.

**Figure 8:**
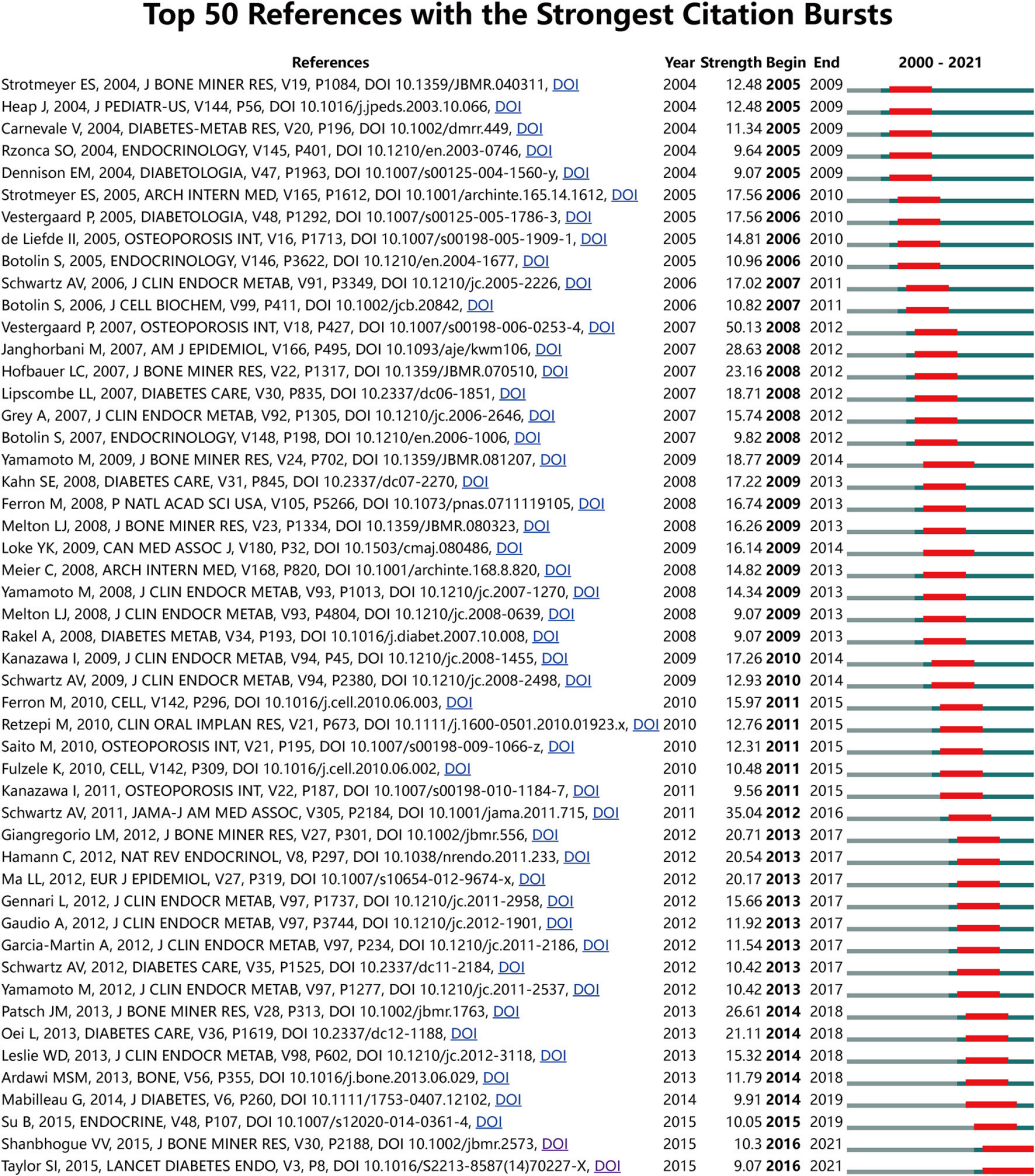
Top 50 references with the strongest citation bursts. The bars in red stands for a burst period for the references.

Table 3 summarizes the characteristics of the top 10 highly cited literature in the research scope of BMDM. The majority of the studies were published before 2010. Of these, three papers were cited over 500 times with all the top 10 cited 370 times or more. Besides that, reference co-citation analysis was also conducted by CiteSpace. As shown in Figure 7 and Supplementary Table 1, all the nodes representing references in the co-citation network map could be clustered into 17 specific clusters. The modularity was 0.89, and the mean silhouette value was 0.9618, reflecting the rationality of this clustering method. All these clusters were generalized and ordered by the number of co-cited references. The first cluster was “#0 glycation end-product,” followed by “#1 oxidative stress” and “#2 energy metabolism.” Apart from that, the references with strong citation bursts were explored via CiteSpace, and the top 50 references with the strongest citation bursts were identified. As indicated in Figure 8, the strongest burst starting from 2008 was from the paper published by Vestergaard P and colleagues in 2007, followed by Schwartz et al. (47), Janghorbani et al. (39).

**Table 3:** Characteristics of top 10 highly cited literatures on BMDM.

**Ranking**	**Title**	**Total citations**	**Average citation frequency per year**	**Journal**	**References**
1	Discrepancies in bone mineral density and fracture risk in patients with type 1 and type 2 diabetes—a meta-analysis	1,248	78	Osteoporosis International	(4)
2	Systematic review of type 1 and type 2 diabetes mellitus and risk of fracture	796	49.75	American Journal of Epidemiology	(39)
3	Osteocalcin differentially regulates beta cell and adipocyte gene expression and affects the development of metabolic diseases in wild-type mice	674	44.93	Proceedings of the National Academy of Sciences of the United States of America	(40)
4	Older women with diabetes have an increased risk of fracture: a prospective study	557	25.32	Journal of Clinical Endocrinology & Metabolism	(41)
5	Collagen cross-links as a determinant of bone quality: a possible explanation for bone fragility in aging, osteoporosis, and diabetes mellitus	539	41.46	Osteoporosis International	(42)
6	Type 1 and type 2 diabetes and incident hip fractures in postmenopausal women	410	18.64	Diabetes Care	(43)
7	Association of BMD and FRAX Score with Risk of Fracture in Older Adults with Type 2 Diabetes	405	33.75	Jama-Journal of the American Medical Association	(41)
8	Mechanisms of diabetes mellitus-induced bone fragility	388	64.67	Nature Reviews Endocrinology	(44)
9	Rosiglitazone-associated fractures in type 2 diabetes—An analysis from a diabetes outcome progression trial (ADOPT)	388	25.87	Diabetes Care	(45)
10	Relative fracture risk in patients with diabetes mellitus, and the impact of insulin and oral antidiabetic medication on relative fracture risk	370	20.56	Diabetologia	(46)

*Ranking: based on the number of total citations*.

### Analysis of Co-occurring Keywords and Related Genes

**Figure 9:**
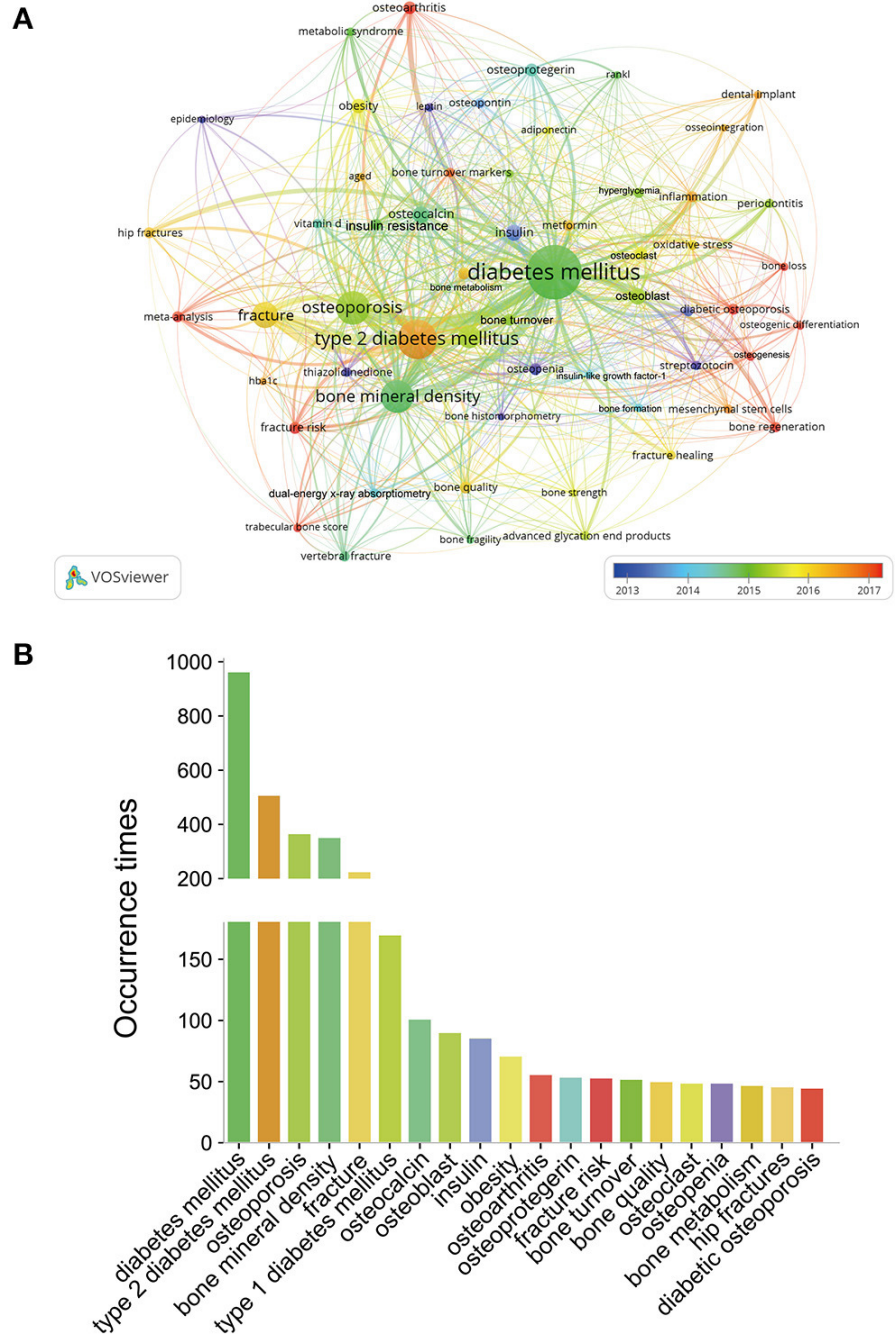
**(A)** Overlay visualization map of keywords analysis in BMDM field based on the VOSviewer. The node size is proportional to the sum of occurrence times. The color of each node implies the average appearing year according to the color gradient in the lower right corner. The blue color represents the keywords appeared relatively earlier, and the dark red color reflects the recent occurrence. **(B)** Frequency distribution of the top 20 most frequent occurrences keywords.

**Figure 10:**
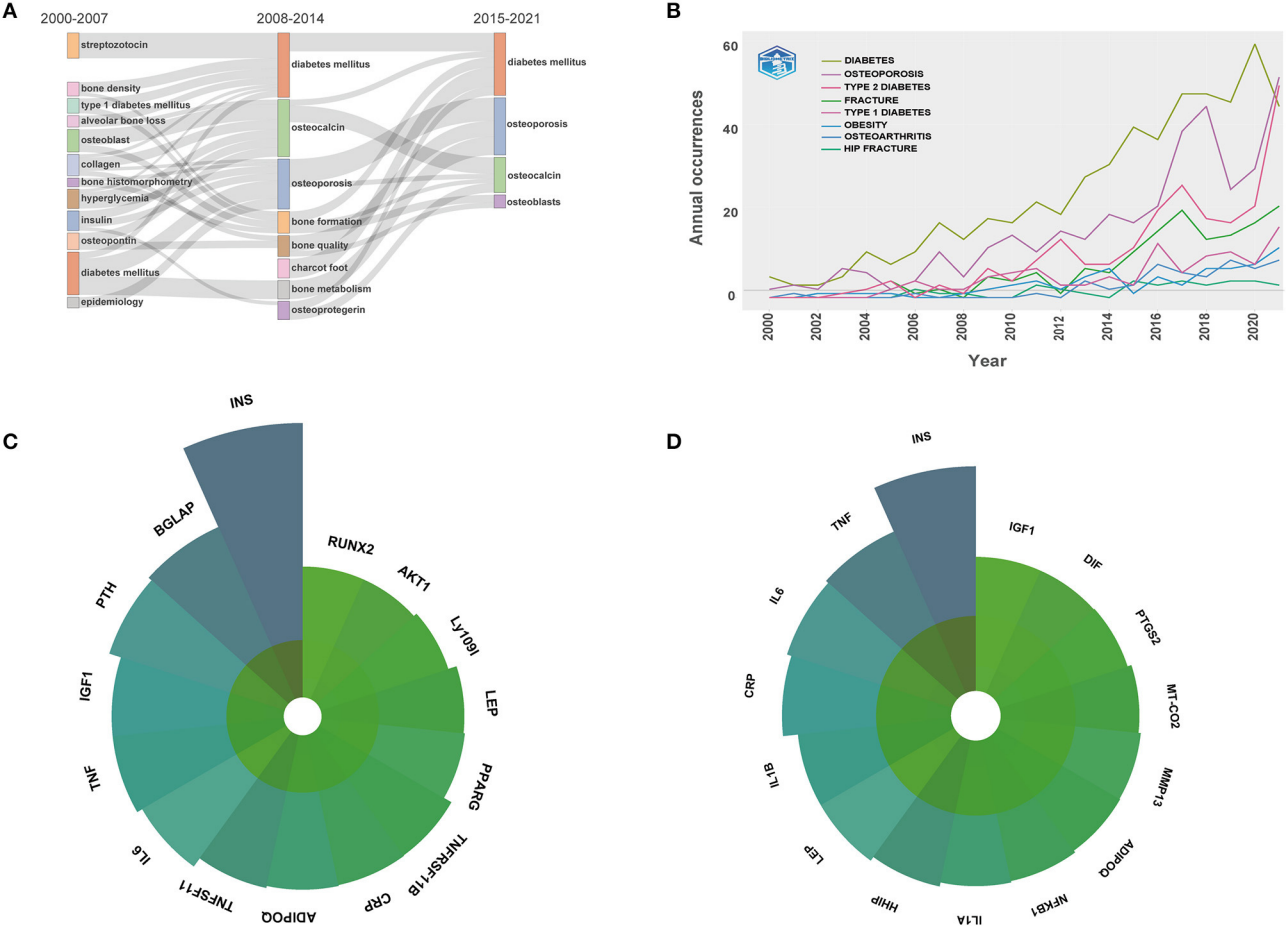
**(A)** Thematic evolution of the three stages in the BMDM research field. The line between nodes reflects the research topic's evolving focus. The width indicates the amount of shared keywords. The thicker the line, the greater the significance of the two subjects. **(B)** The change pattern of annual occurrences frequency of author keywords related to disease. **(C)** Top 15 most studied genes between osteoporosis and diabetes mellitus. **(D)** Top 15 most studied genes between osteoarthritis and diabetes mellitus.

After manual merging the keywords with the same meaning, a total of 58 author keywords with a minimum of 20 occurrences were extracted from the 2,525 publications, and an overlay visualization map was created (Figure 9A). All these keywords were marked with different colors based on AAY, which could reflect the research hotspots in different periods. Figure 9B presents the frequency distribution of the top 20 most frequent occurrences keywords. Among them, the top 5 keywords were as follows: diabetes mellitus (964), type 2 diabetes mellitus (509), osteoporosis (366), bone mineral density (353), and fracture (225). In addition, thematic evolution analysis was also performed by Bibliometrix. Sankey diagram was used to interpret the thematic evolution of three stages in the BMDM research (Figure 10A). The change pattern of annual occurrences frequency of author keywords related to diseases such as diabetes, osteoporosis, osteoarthritis, etc. from 2000 to 2021 is illustrated in Figure 10B. Moreover, Figure 10C illustrates the top 15 most studied genes between osteoporosis and DM, and Figure 10D presents the top 15 genes between osteoarthritis and DM.

## Discussion

### Global Publication Trends in BMDM Research

The change in the amount of academic publications is a vital indicator of the development trend in a field (48). As can be seen from the curve fitting results, the number of publications exhibited an overall rapid growth trend. From the point of view of various stages of development, from 2000 to 2006, the number of scientific publications investigating the links between BM and DM was still very low (no more than 50 publications) and unstable. The study on BMDM was still at an early stage of development, suggesting that the role of bone metabolic aberration occurred in DM did not attract too much attention of medical community at that time. Interestingly, the published studies on BMDM had a steady rise in 2007–2014, and the number has exceeded 100 since 2012. Between 2015 and 2021, the annual publication has drastically increased, and almost 62.7% of them (1,585 papers) were published over the last 7 years. This result may suggest that BMDM has generated increasing attention and interest over recent years. And on this basis, one can predict that the number of publications in this field will further grow with in-depth study of the molecular mechanism and the conduct of clinical experiments on anti-diabetic medicines (44, 49).

### General Knowledge Structures and Major Contributors

#### Countries and Institutions

The total number of literature by a country serves as an important indicator to reflect a country's output and productivity. The results depicted in Table 1 show that countries from North America, Europe, and Asia almost accounted for the top 20 contributing nations on BMDM. Among them, we can see that the United States, China, and Japan are the leading countries where BMDM research is occurring. In the meantime, the United States has the highest H-index. The above results reflect that the United States has made tremendous contribution and established its leading position in the domain of BMDM research. In addition to the BMDM research subject, earlier bibliometric studies revealed that the United States also had the highest number of publications in other areas of BM or DM researches (31, 32, 34). As for countries or institutions collaboration analysis, it can be seen from Figure 3B that the United States collaborated most closely with China, Italy, and Canada. However, when it comes to an institutional level, there were only three institutions with a BC value more than 0.1. BC is an indicator to assess the importance of nodes in a collaboration network. Generally, BC value >0.1 is regarded as vital node (22). From Figures 4A,B, there were relatively few collaboration and exchange of findings among institutions from different countries, and most collaborating institutions were limited to the domestic level. Considering the constant increase in the incidence of DM worldwide, this situation greatly impedes the development of the research field. It is worth noting that, consistent with other bibliometric studies, a lack of cross-institutional cooperation seems to be a general phenomenon in the field of diabetes (50, 51). Therefore, we strongly recommend that institutions from different countries should remove academic barriers and strengthen the cooperation to boost the development of BMDM research.

#### Authors

Among the top 5 most productive authors, Schwartz AV from University of California San Francisco contributed the most articles, followed by Sugimoto T and Kanazawa I from Shimane University. In addition, as can be seen from Figures 5A,B, Schwartz AV was also the author with the highest H-index and average citations per document, and remained high scientific influence in this field from 2000 to 2021. Schwartz et al. mainly focused on the increased fracture risk in diabetic elderly men and women. In 2001, by analyzing the data from 9,654 women aged 65 or older, they have determined diabetes was a risk factor for proximal humerus, hip, and foot fractures in older women. And this study has gained enormous attention and was cited more than 550 times so far (41). Moreover, in another prospective observational study published in *JAMA*, they found that bone mineral density (BMD) T-score of the femoral neck and WHO Fracture Risk Algorithm (FRAX) score were both associated with fracture risk in older adults with T2DM. Nevertheless, compared with these patients without DM, a given T-score or FRAX score was associated with higher fracture risk in older adults with DM (47). This finding has confirmed the useful of BMD T-score and FRAX score for clinical evaluation of fracture risk, and prompted a series of subsequent studies (52, 53).

As for author co-citation analysis, 73 authors with at least 100 citations were included. As displayed in Figure 5D, the top 3 authors with the greatest TLS were Schwartz AV, Vestergaard P, and Janghorbani M. The TLS indicates the impact of documents from an author on other authors involved in these studies. This result further confirms that Schwartz AV was the most influential author. At the same time, it is worth noting that albeit with not large number of publications, Vestergaard P from Aarhus University and Janghorbani M from Isfahan University Medical Science still occupied core locations in the co-citation map. This could have been relevant to several highly-cited publications by them. Unsurprisingly, three studies of them were the top 10 most highly-cited articles in this domain (4, 39, 46). And the study by Vestergaard (4) titled “Discrepancies in bone mineral density and fracture risk in patients with type 1 and type 2 diabetes—a meta-analysis” was the only one study that received more than 1,200 citations in this field. This result suggests that the number of publications may not definitely reflect the academic influence of an author, since there are many factors that influence the citation frequencies of an article (27).

#### Journals

Journals are an important vehicle for presenting the results of academic information and knowledge dissemination. Few researchers are able to fully understand all relevant journals in their field, and many of them struggle to select the most proper outlet and target journals for their studies. By summarizing the basic information drawing the co-citation visual network of the most productive and high-impact journals, the most relevant journals in the research field of BMDM could be identified so that researchers can choose the most suitable journals for submitting their manuscripts. Among the top 20 most productive journals, most relevant studies were published in Q1 or Q2 journals, and *Bone, Osteoporosis International, Journal of Clinical Endocrinology Metabolism, Calcified Tissue International*, and *Journal of Bone and Mineral Research* occupy the first five positions, making them zone core and popular journals in BMDM field. In term of co-citation analysis, the top 5 co-cited journals were *Journal of Bone and Mineral Research, Osteoporosis International, Bone, Journal of Clinical Endocrinology Metabolism, Diabetes Care*. Research findings related to BMDM that published in these journals may have great potential to be cited and receive more attention. Therefore, scholars may consider these priority journals in the future, and scientific outputs by these journals also deserve special attention to obtain recent advances in this domain.

### Research Clusters and Research Focus Transition

Reference and keyword analysis is one of the key methodologies and most significant indicators in bibliometrics. Based on the reference co-citation analysis and keyword co-occurrence analysis, the main research directions, hotspots, and evolution process in the field can be uncovered (54, 55).

#### References Co-citation Analysis

Using the CiteSpace software, a visualization network map of co-cited references was generated in Figure 7. All these 17 BMDM research clusters in the past 22 years were ordered from the largest to smallest based on the number of co-cited references. As can be seen from Supplementary Table 1, “glycation end-product” was the largest cluster (#0), followed by “oxidative stress” (#1), and “energy metabolism” (#2). In addition, the timeline chart could cluster references and take time into account, which is convenient for us to understand the period of a particular topic and explore the evolution track of this field (34). According to mean year of these clusters, we can see that the research hotspots have shifted to “high glucose environment” (#8), “glycation end-product” (#0), and “sodium-glucose co-transporter” (#7).

#### High Glucose Environment

Although glucose represent key molecules in cellular energy metabolism, previous studies revealed that hyperglycemia had an adverse effect on bone quality. *In vitro* experiments have proven that high glucose environment exerts an inhibitory action on the osteogenic differentiation and growth of osteoblasts and bone marrow stroma cells (BMSCs) (18, 56). Glycolysis is a fundamental metabolic pathway for glucose catabolism. However, under a high-glucose microenvironment, excess glucose may activate other metabolic pathways such as the polyol pathway, the protein kinase C signaling pathway, the formation of glycation end-products, and the hexosamine pathway, all of which induce the accumulation of oxidative stress and the production of inflammatory cytokines (57, 58). Furthermore, there is also study showing that sustained hyperglycemia may trigger cellular innate immune responses, activate reactive oxygen species (ROS)-producing enzymes localized on the outer mitochondrion, resulting in overproduction of ROS and cell dysfunction (59). Therefore, in recent years, a large number of studies have focused on how to rescue the impaired osteogenesis differentiation ability of BMSCs and osteoblasts (56, 57, 60, 61). That is very critical and meaningful way to find novel therapeutic targets for diabetic osteoporosis.

#### Glycation End-Product

Advanced glycation end products (AGEs) are irreversibly produced from non-enzymatic glycation and oxidation of nucleic acids, lipids and proteins. The receptors for AGEs (RAGE) are the main receptors involved in the cellular uptake and degradation of AGEs and are also known as a pattern recognition receptor. Previous studies revealed that RAGE and its ligands play important roles in bone homeostasis (62, 63). RAGE knockout mice exhibit decreased bone resorptive activity and increased bone mineral density (62). AGEs were reported to suppress osteogenic differentiation of adipose-derived stem cells (ASCs) via interfering with the Wnt/β-catenin signaling pathway (63). In addition, accumulation of AGEs could also promote expression of transforming growth factor (TGF)-β and IL-6 to induce apoptosis of osteoblasts and elevate osteoclasts activity (64, 65). As previously stated, protracted exposure to excessive blood glucose results in excessive formation and accumulation of AGEs. Increasing evidence indicates that the accumulation of AGEs are important contributors of diabetic bone loss (13). Accordingly, small-molecular inhibitors such as TTP488, FPS-ZM1, etc. targeting RAGE and its intracellular signaling pathway have been developed (66, 67). These small molecule inhibitors hold great promise for the treatment of multiple metabolic bone diseases and merit further investigation.

#### Sodium-Glucose Co-transporter

Sodium glucose cotransporter-2 (SGLT2) is a sodium-dependent glucose transporter involved in glucose reabsorption in the kidney. The inhibitors of SGLT2 have been established as a novel therapeutic approach for diabetic control, working by decrease the reabsorption of glucose and increase the glucose excretion. Since the first SGLT-2 inhibitor, canagliflozin, approved by USA Food and Drug Administration in 2013, more than seven SGLT-2 inhibitors such as empagliflozin, ertugliflozin and dapagliflozin, etc. have been introduced into the clinic nowadays (68). However, there remains controversy about the effects of different SGLT-2 inhibitors on bone metabolism. Among them, canagliflozin is the most studied. In the Canagliflozin Cardiovascular Assessment Study (CANVAS) trial, an increased risk of fractures was observed in patients treated with canagliflozin, which triggered concerns about bone health (69). The exact mechanisms of canagliflozin increasing fracture risk are unclear, and existing evidence suggests that canagliflozin might have an impact on the homeostasis of calcium and phosphate, and the secretion of parathyroid hormone (PTH) (70). Despite all of this, multiple subsequent studies have found that canagliflozin was not associated with an increased risk of fractures (71, 72). Therefore, available evidence on SGLT-2 inhibitors is somehow conflicting and cannot fully support a direct responsibility for bone fractures. In addition, the effects of other kinds of SGLT-2 inhibitors on bone health need more investigations to confirm (73).

#### References With Citation Burst

Apart from cluster analysis, burst references are considered another important indicator to track and capture the research hotspots and emerging trends over time. Burst references refers to references heavily cited by other studies over a period of time, which implies that they have received particular attention at a certain time period (34). References with outbreak durations ≥5 years were shown in Figure 8. Top 50 references with the strongest citation bursts were listed. Among these references, the strongest burst starting from 2008 was from the paper published by Vestergaard P in 2007 (4), followed by Schwartz et al. (47), Janghorbani et al. (39). Additionally, references with citation bursts were first appeared in 2005 due to 5 literatures published in 2004 (74–78). Of these, a multiethnic study conducted by Strotmeyer et al. found that T2DM was associated with higher BMD in all race-gender elderly adults, and independent of central adiposity, increased obesity, or fasting insulin levels. Heap et al. (75) investigated whether blood glucose regulation and disease duration could influence the bone characteristics in adolescents with T1DM. Their findings suggest that altered BMD acquisition in these adolescents might limit the acquisition of peak bone mass and increase the risk of developing osteoporosis in later life. An elegant review by Carnevale et al. (76) summarized the evidence that confirmed the diabetic patients with higher fracture risk and the possible mechanisms. While Rzonca et al. (77), found that rosiglitazone therapy posed potential risks of adverse skeletal effects based on *in vitro* experiments. Regarding the fifth research, their study confirmed the positive association between BMD and T2DM, and partly mediated by adiposity (78). Of note, there are still two references published in 2015 with ongoing burst, which means they have received substantial attention recently (70, 79). Of them, one cross-sectional *in vivo* study has assessed the bone characteristics in adult T1DM patients with and without diabetic microvascular disease. Their findings suggested that the presence of diabetic microvascular disease was associated with the deficits in bone microarchitecture. Another study by Taylor et al. (70) explored the possible downstream mechanisms of SGLT2-inhibitors on bone. This finding illustrates again that SGLT2 is a hot topic in BMDM research.

#### Keywords Analysis and Related Genes

Author keywords are usually the most highly represented terms selected to explain the subject matter of research (34). In this study, we constructed the keyword co-occurrence network map with VOSviewer software. As several author keywords had various forms, but the same meaning. After manual merging the keywords with the same meaning, a total of 58 author keywords were extracted from the 2,525 publications, and an overlay visualization map was created in Figure 9A. Besides the keywords related to DM, osteoporosis and related fractures and osteoarthritis are the main bone metabolic diseases of greatest concern in this field. In the meantime, it can be seen that although patients with T1DM have a much higher risk of fracture compared to those with T1DM, researchers' concern about T2DM is relatively higher, which may have relevance to the higher incidence of T2DM. Moreover, Sankey diagram of authors' keywords was conducted to interpret the thematic change and evolution in the BMDM research field. Several popular research topics in a certain period may slow down with the appearance of other novel directions (80). The period of 22 years considered for our included publications was split into 3 periods: 2000–2007, 2008–2014, and 2015–2021. In the first period, thematic evolution was observed in 12 research areas: streptozotocin, bone density, type 1 diabetes mellitus, alveolar bone loss, osteoblast, collagen, bone histomorphometry, hyperglycemia, insulin, osteopontin, diabetes mellitus, epidemiology. As for the second period, seven new thematic domains including osteocalcin, osteoporosis, bone formation, bone quality, charcot foot, bone metabolism, and osteoprotegerin emerged. When it comes to the third period, the research topics were more centrally and diabetes mellitus and osteoporosis remain as the primary themes.

In the overlay visualization map of keyword co-occurrence analysis, different keywords were marked with various colors based on AAY. The blue color represents the keywords appeared relatively earlier, and the dark red color reflects the recent occurrence. Thus, these keywords with blue color such as “insulin,” “leptin,” “epidemiology,” and “streptozotocin” were the major topics during the early stage. And these keywords such as “diabetic osteoporosis,” “osteoarthritis,” “fracture risk,” “meta-analysis,” “osteogenic differentiation,” “bone regeneration,” “osteogenesis,” and “trabecular bone score” (81) were colored in red, which suggests that these research topics are attracting attention recently and may remain the research hotspots and frontiers in the near future. Take meta-analysis as an example, it is an important tool for evidence-based medicine. In previous studies, scholars have conducted extensive meta-analyses to assess the relationship between BMD/bone loss/fracture and DM/antidiabetic medication (4, 82, 83). Of these, the most well-known one was conducted by Vestergaard (4). However, due to the lack of large-sample randomized controlled studies in BMDM field, most of meta-analysis were based on retrospective studies. Further randomized controlled trials of high quality are warranted to be performed for update of the results of these meta-analyses.

Moreover, as osteoporosis and osteoarthritis are the most representative metabolic bone diseases associated with DM, we also summarized the most studied genes among them by online data analysis website. As shown in Figures 10C,D, *INS, BGLAP, PTH, IGF1*, and *TNF* were the top 5 most studied genes between osteoporosis and DM (84, 85). As for osteoarthritis and DM, the top 5 most related genes were *INS, TNF, IL-6, CRP*, and *IL-1B* (86–88). It can be seen that, unlike diabetic osteoporosis, multiple inflammatory factors are involved in the progress of diabetic osteoarthritis. Recently, increasing studies have uncovered that in diabetic patients with osteoarthritis, diabetic treatment such as DPP-4 could partially improve osteoarthritis symptoms by decreasing the production of inflammatory cytokines such as IL-6, IL-8, and TNF-α (86, 87). These results could serve as a reference for investigators in the field.

## Strengths and Limitation

The present study has certain strong points in contrast to previous studies that adopted only meta-analysis or narrative reviews. Most importantly, it is the first bibliometric study to map and characterize the knowledge landscapes on BMDM from 2000 to 2021. Meanwhile, we used multiple types of bibliometric software and tools for analysis and visualization, which would add richness to the results. Another strength is checking the quality of literature included in the final analysis. However, there are also several additional limitations to our work. First of all, data on BMDM were primarily retrieved and collected from the WoSCC database, which would miss several related publications not included in these databases. However, it should be noted that the WoSCC is the most commonly used database for bibliometric analysis, and the data from WoSCC can represent the condition of most publications in a certain field to some extent. There have been few studies used more than two electronic databases in the previous studies owing to the limitation of file formats (22, 23, 36). Secondly, despite our manual screening and normalization procedures, selection bias may have still existed due to the merge of some keywords and continuous updates of the database. Nevertheless, we believe that our findings are still to be an effective representation of the global outputs of bone metabolism in diabetes mellitus. Finally, we only included literature published in English, meaning that some relevant publications may have been missed.

## Conclusions

This study provides a missing analysis of global research progress on diabetes mellitus and bone metabolism. Analysis of all literature published in English showed an overall increasing trend. Major contributions were from North American, European, and Asian countries, institutions, and authors, led by the United States. The journals that published the most BMDM-related papers were *Bone* and *Osteoporosis International*. Osteoporosis and related fractures are the main bone metabolic diseases of greatest concern in this field. The main funding agencies and collaborations were also found to be from developed regions, showing that increased collaboration is needed to boost the development of BMDM research. Several research topics including high glucose environment, glycation end-product and sodium-glucose co-transporter have been recognized as the current research focus in this domain. The following research directions such as diabetic osteoporosis, osteoarthritis, fracture risk, meta-analysis, osteogenic differentiation, bone regeneration, osteogenesis, and trabecular bone score may remain the research hotspots and frontiers in the near future. All in all, we believe that this bibliometric study can help researchers explore potential cooperation opportunities, and also understand BMDM field's knowledge landscapes, evolution process, and research hotspots in this field. This study represents a call to researchers and clinicians that like other diabetic complications, diabetic osteopenia deserves more attention.

## Data Availability Statement

The original contributions presented in the study are included in the article/Supplementary Material, further inquiries can be directed to the corresponding author/s.

## Author Contributions

KC, ZS, and HW designed the study. KC, QG, ZS, and WY collected the data. KC, WY, YW, and HW analyzed the data and drafted the manuscript. KC, ZS, WY, and QG revised and approved the final version of the manuscript. All authors read and approved the submitted version.

## Funding

This work was supported by the Tianjin Municipal Health Bureau (grant number 14KG115) and Key Program of the Natural Science Foundation of Tianjin (grant number 20JCZDJC00730).

## Conflict of Interest

The authors declare that the research was conducted in the absence of any commercial or financial relationships that could be construed as a potential conflict of interest.

## Publisher's Note

All claims expressed in this article are solely those of the authors and do not necessarily represent those of their affiliated organizations, or those of the publisher, the editors and the reviewers. Any product that may be evaluated in this article, or claim that may be made by its manufacturer, is not guaranteed or endorsed by the publisher.

## Abbreviations

DM: diabetes mellitus
WHO: World Health Organization
T2DM: type 2 DM
BM: bone metabolism
T1DM: type 1 DM
CMA: Chinese Medical Association
BMDM: bone metabolism and diabetes mellitus
DPP-4: peptidase-4
WoSCC: Web of Science Core Collection
SCIE: Science Citation Index Expanded
TI: titles (TI)
AK: author keywords
JIF: journal impact factor
JCR: Journal Citation Report
AAY: average appearing year
BC: betweenness centrality
TLS: total link strength
BMD: bone mineral density
FRAX: WHO Fracture Risk Algorithm
BMSCs: bone marrow stroma cells
ROS: reactive oxygen species
AGEs: advanced glycation end products
RAGE: receptors for AGEs
ASCs: adipose-derived stem cells
SGLT2: Sodium glucose cotransporter-2
CANVAS: Canagliflozin Cardiovascular Assessment Study
PTH: parathyroid hormone.
