# Supplementary material for: Mapping Knowledge Landscapes and Emerging Trends of the Links Between Bone Metabolism and Diabetes Mellitus: A Bibliometric Analysis From 2000 to 2021
Source: Front Public Health. 2022 Jun 3;10:918483. doi: 10.3389/fpubh.2022.918483 (PMC9204186; doi:10.3389/fpubh.2022.918483)
Supplement: Supplementary file 1 [file Data_Sheet_1.pdf]

Supplementary Material

1 Supplementary Figures and Tables

1.1 Supplementary Figure

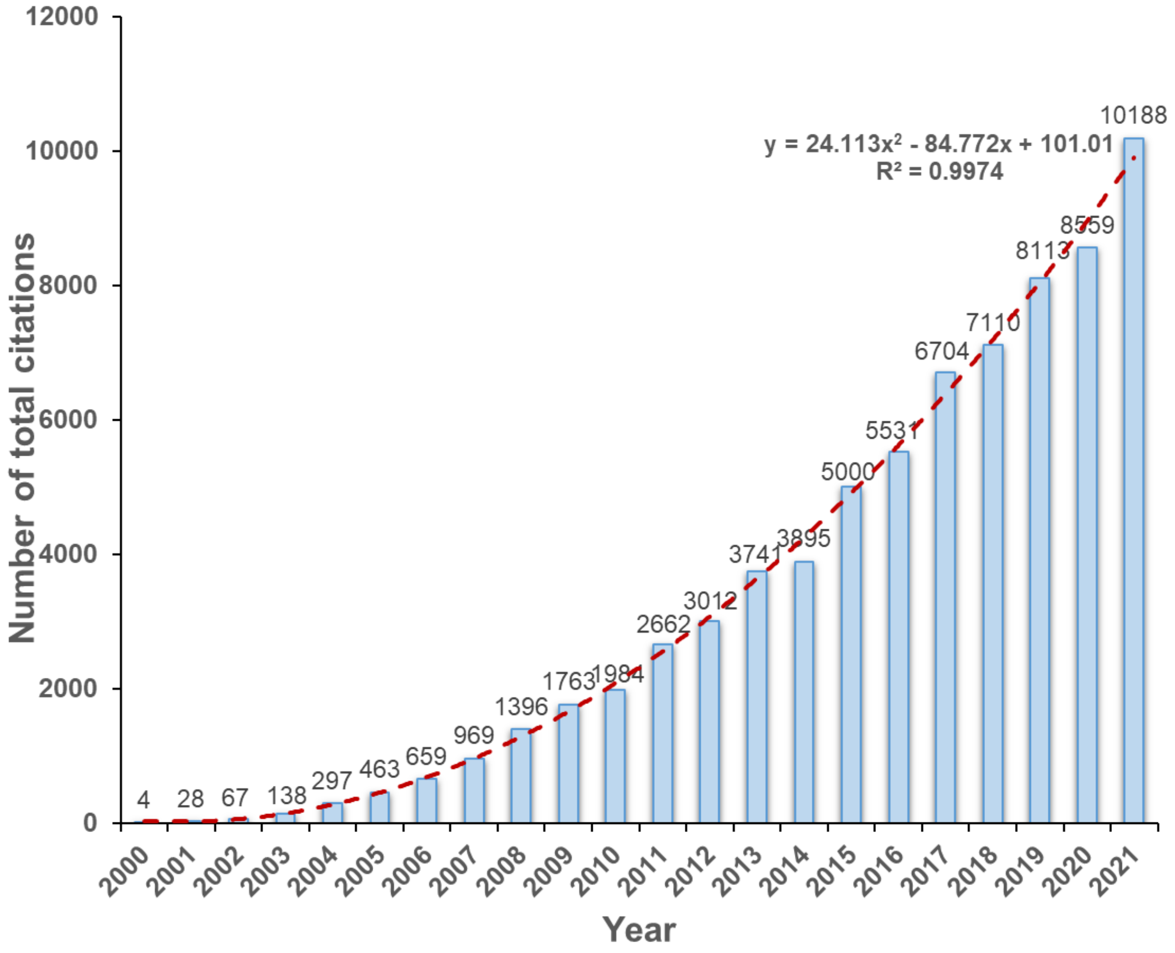

Supplementary Figure 1. Distribution of the annual number of citations regarding bone metabolism in diabetes mellitus research from 2000 to 2021.

Supplementary Table 1. The clusters information of co-cited references

Cluster ID	Size	Mean (Year)	Silhouette	Label (LLR algorithm)
#0	113	2016	0.883	**glycation end-product**; elderly women; bone microarchitecture; bone material strength; bone material properties
#1	99	2005	0.885	**oxidative stress**; diabetic bone loss; diabetic bone phenotype; accelerated loss; diabetes severity
#2	97	2008	0.915	**energy metabolism**; serum osteocalcin level; metabolic bone disease; new link; evolutionary clue
#3	91	2000	0.941	**body composition study**; health aging; tromso study; bone characteristics; black adult
#4	81	2006	0.949	**clinical management**; diabetes treatment; longitudinal risk; pioglitazone increase fracture risk; diabetes tzd
#5	78	2012	0.841	**fracture outcome**; type-2 diabete; skeletal metabolism fracture risk; early process; diabetes-associated bone alteration
#6	76	2014	0.857	**trabecular bone score**; low-energy fracture; non-vertebral fracture; bone strength; cohort studies
#7	71	2015	0.851	**sodium-glucose co-transporter**; sodium glucose co-transporter; fracture risk; controlled trial; cardiovascular benefit
#8	65	2017	0.906	**high glucose environment**; italian society; mesenchymal stem cell; bone marrow; osteogenic differentiation
#9	64	2014	0.931	**peptidase-4 inhibitor**; bone metabolism; other anti-hyperglycemic drug; glucagon-like peptide-1 receptor agonist; diabetes therapy
#10	51	2011	0.849	**stromal cell**; bone defect regeneration; diabetes medication; bone metabolism; anti-osteoporotic effect
#11	50	2005	0.989	**serum osteoprotegerin level**; diabetic macroangiopathy; diabetic nephropathy; osteoprotegerin serum release; endothelial cell dysfunction
#12	38	1997	0.996	**non-diabetic men**; anabolic function; catabolic function; insulin receptor substrate-2; insulin-dependent diabetes mellitus
#13	33	2010	0.924	**diabetic fracture healing**; charcot neuroarthropathy; molecular perspective; life signal; diabetes-derived osteoclast
#14	32	2000	0.99	**ok rat**; diabetic metabolic state; delayed remodeling; early period; biomechanical abnormalities

#15	18	2014	0.986	**osteoarthritis initiative**; knee osteoarthritis; biochemical knee cartilage composition; diabetes-induced osteoarthritis; human synovial fluid
#16	12	2000	1	**patient**; type; hmg-coa reductase inhibitor; atorvastatin; marker
